# Factors associated with health status and exacerbations in COPD maintenance therapy with dry powder inhalers

**DOI:** 10.1038/s41533-022-00282-y

**Published:** 2022-05-26

**Authors:** Janwillem W. H. Kocks, Hans Wouters, Sinthia Bosnic-Anticevich, Joyce van Cooten, Jaime Correia de Sousa, Biljana Cvetkovski, Richard Dekhuijzen, Lars Dijk, Evgeni Dvortsin, Marina Garcia Pardo, Asparuh Gardev, Radosław Gawlik, Iris van Geer - Postmus, Iris van der Ham, Marten Harbers, Alberto de la Hoz, Ymke Janse, Marjan Kerkhof, Federico Lavorini, Tiago Maricoto, Jiska Meijer, Boyd Metz, David Price, Miguel Roman-Rodriguez, Kirsten Schuttel, Nilouq Stoker, Ioanna Tsiligianni, Omar Usmani, Marika T. Leving

**Affiliations:** 1 General Practitioners Research Institute, Groningen, The Netherlands; 2 University of Groningen, University Medical Center Groningen, GRIAC Research Institute, Groningen, The Netherlands; 3 Observational and Pragmatic Research Institute, Singapore, Singapore; 4 Department of Pulmonology, University of Groningen, University Medical Center Groningen, Groningen, The Netherlands; 5 Woolcock Institute of Medical Research, University of Sydney, Sydney, Australia; 6 Sydney Local Health District, Sydney, Australia; 7 Life and Health Sciences Research Institute (ICVS), PT Government Associate Laboratory, School of Medicine, University of Minho, Braga, Portugal; 8 Radboud University Medical Center, Nijmegen, The Netherlands; 9 Primary Care Respiratory Research Unit, Instituto De Investigación Sanitaria De Baleares (IdISBa), Palma de Mallorca, Spain; 10 Boehringer Ingelheim International GmbH, Ingelheim am Rhein, Germany; 11 Department of Internal Medicine, Allergology, Clinical Immunology, Medical University of Silesia, Katowice, Poland; 12 Department of Clinical and Experimental Medicine, Careggi University Hospital, Florence, Italy; 13 Faculty of Health Sciences, University of Beira Interior, Covilha, Portugal; 14 Centre of Academic Primary Care, Division of Applied Health Sciences, University of Aberdeen, Aberdeen, UK; 15 Department of Social Medicine, Health Planning Unit, Faculty of Medicine, University of Crete, Rethymno, Greece; 16 Airway Disease, National Heart and Lung Institute (NHLI), Imperial College London and Royal Brompton Hospital, London, UK

**Keywords:** Chronic obstructive pulmonary disease, Outcomes research

## Abstract

The study aimed to determine the associations of Peak Inspiratory Flow (PIF), inhalation technique and adherence with health status and exacerbations in participants with COPD using DPI maintenance therapy. This cross-sectional multi-country observational real-world study included COPD participants aged ≥40 years using a DPI for maintenance therapy. PIF was measured three times with the In-Check DIAL G16: (1) typical PIF at resistance of participant’s inhaler, (2) maximal PIF at resistance of participant’s inhaler, (3) maximal PIF at low resistance. Suboptimal PIF (sPIF) was defined as PIF lower than required for the device. Participants completed questionnaires on health status (Clinical COPD Questionnaire (CCQ)), adherence (Test of Adherence to Inhalers (TAI)) and exacerbations. Inhalation technique was assessed by standardised evaluation of video recordings. Complete data were available from 1434 participants (50.1% female, mean age 69.2 years). GOLD stage was available for 801 participants: GOLD stage I (23.6%), II (54.9%), III (17.4%) and IV (4.1%)). Of all participants, 29% had a sPIF, and 16% were shown able to generate an optimal PIF but failed to do so. sPIF was significantly associated with worse health status (0.226 (95% CI 0.107–0.346), worse units on CCQ; *p* = 0.001). The errors ‘teeth and lips sealed around mouthpiece’, ‘breathe in’, and ‘breathe out calmly after inhalation’ were related to health status. Adherence was not associated with health status. After correcting for multiple testing, no significant association was found with moderate or severe exacerbations in the last 12 months. To conclude, sPIF is associated with poorer health status. This study demonstrates the importance of PIF assessment in DPI inhalation therapy. Healthcare professionals should consider selecting appropriate inhalers in cases of sPIF.

## Introduction

COPD is a chronic and progressive lung disease, impacting the lives of ~384 million patients worldwide^1,2^. The main pharmacotherapeutic treatment for COPD is maintenance therapy with long-acting bronchodilators^3^. The most used devices for administration of long-acting bronchodilators are dry powder inhalers (DPIs)^4^.

Despite the variety of available therapies, studies reveal that COPD tends to be undertreated^5^. This may be partially explained by the incorrect use of inhalers, which results in critical errors, a limitation of the dose of medication that is delivered into the airways and eventually, undertreatment. This means that even if the patient is adherent to prescribed treatment regimens, a clinical response may not be sufficiently achieved^6^. A successful treatment of COPD with maintenance therapy depends on a complex constellation of factors, among which all aspects regarding breathing manoeuvres.

Taking into consideration that DPIs are breath-actuated devices, it is crucial that patients can generate a sufficient peak inspiratory flow rate (PIF) to enable optimal drug delivery to the airways^7–9^. The relation between PIF and drug delivery is based on the fact that a minimum inspiratory flow is required to de-agglomerate the medication-containing powder from its lactose carrier within the DPI, so that particles of <5 μm in diameter are released from the inhaler device. Several independent predictors of patients being unable to generate an optimal PIF have been identified, such as female gender, shorter height, and older age^10,11^.

Although patients might be able to generate sufficient flow for a given device with maximal effort and attention to technique, this flow is often not generated in daily life^12^. The typical PIF of a patient is defined as the PIF achieved with the inhaler device that the patient uses in everyday life. If the typical PIF is lower than the optimal PIF for a given device, it seems likely that the patient does not generate enough inspiratory flow to overcome the internal resistance of the device. In such cases, medication will not be optimally inhaled, potentially lowering the efficacy of the maintenance therapy. Therefore, it is recommended to select a device with lower internal resistance for patients with a limited ability to generate sufficient PIF. A reduced PIF with a mismatch in chosen inhaler device predicts all-cause and COPD readmissions in patients with COPD^9^.

Another factor that is likely to reduce the efficacy of therapy, is having a poor inhalation technique^13–16^. Commonly made inhalation technique errors by patients with COPD include drug priming without inhalation, failure to exhale before inhalation, exhalation into the inhaler, an inadequate generation of inspiratory flow, lack of chin lift and making multiple inhalations with one actuation^12,17^. In addition to successful inhalation, medication adherence is an important prerequisite for the effectiveness of maintenance therapy^18^. Adherence to prescribed treatments is generally low in primary care patients with COPD^19^. Non-adherence can be categorized as sporadic (e.g. patient forgets to take the medication), deliberate (e.g. due to patient’s perception of medication necessity^20^, but it can also be unconscious (e.g. due to inappropriate handling of the DPI)^21^.

Of particular concern to real-life practice is the fact that, although the majority of patients are treated in primary care in many countries, little is known about the prevalence of suboptimal PIF and/or inhalation technique errors in COPD patients in this healthcare setting. Also, the associations between a suboptimal PIF, inadequate inhalation technique and medication adherence with effectiveness of COPD maintenance therapy remain largely unexamined. PIF and inhalation technique errors are rarely assessed in primary care for the principle aim of evaluating and selecting an inhaler device. Moreover, in COPD, in contrast to asthma, it is unknown whether some inhalation technique errors are more critical than others for the effectiveness of treatment^15,16,22^. In this study, critical errors will be determined based on their association with health status.

Together, all these factors are likely to increase the risk of patients not optimally benefiting from maintenance therapy.

To understand how the interactions of these factors together may negatively affect health status of COPD patients and contribute to the risk of exacerbations, we evaluated this in the PIFotal COPD study. The aim of this study is to determine the association of PIF, inhalation technique errors and adherence with health status and exacerbations in COPD patients receiving maintenance therapy with a DPI.

## Methods

### Study design

**Fig. 1:**
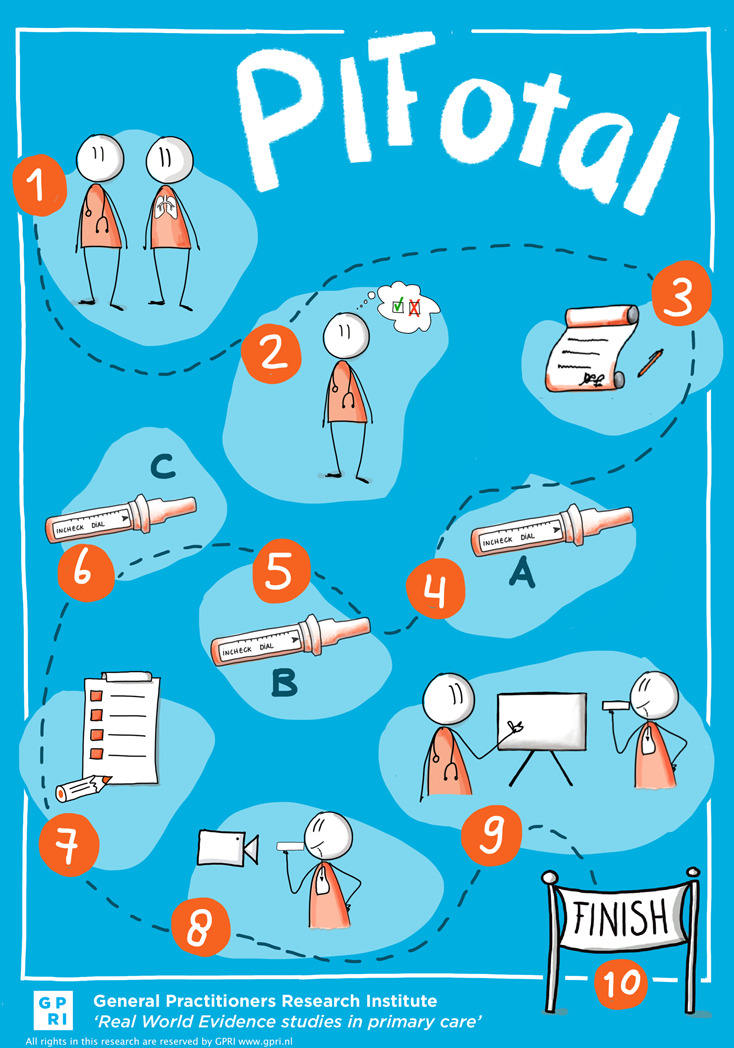
Flow chart of PIFotal COPD study. Participants were invited for clinical examination (Step 1) and eligibility was verified (Step 2). Prior to participation, participants provided written informed consent (Step 3). Subsequently, their PIF was assessed, namely typical PIF (Step 4), a patient’s maximal PIF against the resistance of their own device (Step 5) and a patient’s maximal PIF at low resistance (Step 6). Next, participants filled out questionnaires to assess health status, number of exacerbations, self-reported medication adherence, medication use, and demographic and clinical covariates (Step 7). Subsequently, participants inhaled their usual medication, which was video recorded for later assessment (Step 8). Lastly, participants received tailored inhalation instructions based on the inhalation errors they made (Step 9) after which the clinical assessment was finished (Step 10).

The PIFotal COPD study was a cross-sectional observational real-world study in five European countries (Greece, the Netherlands, Poland, Portugal, Spain) and Australia^23^. Participants were recruited and included in the study between October 2020 and May 2021. PIFotal was registered in a public database prior to execution (clinicaltrials.gov identifier NCT04532853). Local medical ethics committees reviewed and approved the study protocol, and all subjects gave written informed consent. A flow chart of study procedures is illustrated in Fig. 1.

### Study population

Inclusion/exclusion criteria were limited to ensure a real-world setting as much as possible. Participants with a clinical diagnosis of COPD, aged 40 years or older, who were treated with a DPI as maintenance therapy for their COPD in the previous 3 months or longer, were eligible for participation. Participants were excluded from participation if they were unable to give informed consent, were participating in other trials with COPD medication, if they had an exacerbation in the 6 weeks prior to participation or if they had a life-threatening disease with a life expectancy <6 months.

### Peak inspiratory flow (PIF)

PIF (L/min) was assessed with the In-Check DIAL G16 (Clement Clarke, UK), a multi-patient device using disposable, single-patient mouthpieces with inspiratory one-way valves. The In-Check DIAL G16 can be set to resemble resistance of the participant’s inhaler. If a participant used multiple inhalers, the assessment priority determined the typical measurements with PIF and inhaler measurements. The priority assigned to the devices to determine the primary inhaler can be found in Supplementary Table 1.

With the In-Check DIAL G16, PIF was assessed in three ways: (1) typical PIF at the resistance of the own inhaler, (2) maximal PIF at the resistance of the own inhaler and (3) maximal PIF at low resistance^24,25^. For the typical PIF measurement participants were asked to inhale with the In-Check DIAL as they normally would with their DPI. For both maximal PIF measurements, participants were instructed to exhale fully and then inhale as hard and fast as possible. Maximal measurements were performed twice. The maximal of the two attempts was included in the data analysis. A suboptimal PIF was defined as typical PIF being lower than required for the device (Table S1), and optimal PIF as typical PIF equal to or higher than required for the device.

Participants with a typical PIF that was equal to or higher than the optimal PIF for their device were pragmatically named the ‘can and will do’ group. Participants with a typical PIF below the optimal PIF for their device, but able to perform a maximal PIF that is equal to or higher than the optimal PIF, were named the ‘can, but will not do’ group. Participants with both their typical and their maximal PIF below the optimal PIF for their device were named the ‘cannot do’ group.

### Inhalation technique and adherence

Inhalation technique was observed and documented by video recording which was rated offline for errors by two independent observers. They used checklists on inhaler-specific and inhaler-independent commonly made errors, based on recommendations of the Netherlands Lung Alliance (www.inhalatorgebruik.nl) or, if unavailable for specific devices, the Aerosol Drug Management Improvement Team (www.inhalers4u.org). Inhalation technique was evaluated by grouping errors in steps together in 11 categories (Supplementary Table 2). To assess which errors could be regarded as critical in this analysis, all inhalation technique error groups (Supplementary Table 2) were tested for their association with moderate and severe exacerbations and health status (CCQ-score).

Adherence was calculated based on the answers on the 12-item Test of Adherence to Inhalers (TAI-12). Because of the more precise testing of inhalation technique in the study, item 12, concerning physician-observed critical inhalation technique errors, was replaced with the objective assessment of inhalation technique video. Items 1 to 10 could be scored 1–5 points each. Item 11 and inhalation technique could be scored with 1 or 2 points. Only if participants scored the maximum number of points on all items (total 50 points), he or she was considered adherent. We further used three different representations of non-adherence as exploratory predictors: sporadic non-adherence (TAI-12 items 1 to 5 < 25), deliberate non-adherence (TAI-12 items 6 to 10 < 25) and unconscious non-adherence (TAI-12 item 11 and video assessment <4)^26^.

### Health status, exacerbations and healthcare resources

COPD-related health status was measured with the 10-item self-administered CCQ^27^, consisting of three domains: symptoms, functional status and mental health. The CCQ-score is the mean score of 10 item-scores, where each item is scored on a 7-point Likert scale indicating the severity of symptoms. Exacerbations in the previous 12 months were counted from the medical records (32%) or reported by the participants (68%), and were evaluated as either moderate, severe or combined. Moderate exacerbations were defined as exacerbations treated with oral corticosteroids or antibiotics without hospital admission and severe exacerbations were defined as exacerbations requiring hospital admission.

The CAT was self-administered by the participant and consists of 8 items with 5-point Likert scales to rate symptoms (e.g. frequency of coughing), disability, quality of sleep and energy.

### Statistical analysis

The primary objective was to determine the associations of PIF, inhalation technique errors, and overall medication adherence with health status. There were two secondary objectives to determine the associations of PIF, inhalation technique errors, and overall medication adherence with the number of exacerbations (A) and to determine the proportion of participants with suboptimal PIF and different inhalation technique errors for clusters of inhaler devices (according to internal inhaler resistance) (B).

Inhalation technique videos were scored via a list of potential observed technique errors, specified per inhaler. For this article, the relation between observed errors and health status was assessed. We first constructed univariate models, and, to prevent type I statistical errors, inhalation errors with *p*-values < 0.1 were further considered as critical errors in the multi-level models. Three inhalation errors from this list occurred related to CCQ scores with a *p*-value < 0.1 and were further considered as critical errors in the analyses.

For each outcome-predictor combination, a multi-level regression model was fitted, allowing for a random effect at the level of the participants’ general practitioner (*n* = 621). Multiple imputation was used to handle missing data. Each candidate confounder was tested separately for bias potential, defined as the change in coefficient of the fixed effect under study. All candidate confounders were added to the model one by one, sorted by bias potential in a descending order. Whenever the bias potential was ≥5%, the candidate confounder was retained in the model. A list of all candidate confounders can be found in Supplementary Table 3 and an overview of confounders included in the models can be found in Supplementary Table 4.

Since for each outcome measure associations were assessed for 5 variables (suboptimal PIF, overall adherence and 3 critical errors), we adjusted the *p*-values for multiple testing using the false discovery rate according to Simes^28^. All statistical analyses were done using Stata version 15/MP.

### Sample size calculation

A sample size calculation was performed prior to study execution. A sample size of 1200 participants was estimated to be sufficient to achieve sufficient (≥80%) statistical power. It was assumed that the difference between optimal and suboptimal PIF would lead to a Clinical COPD Questionnaire (CCQ) score difference of 0.2 points^29^. This minimal detectable difference yielded a sample size of 1176 participants, with an *α* of 5% and a power of 80%.

### Ethics approval

The PIFotal COPD study protocol received approvals from the following institutional ethics committees/institutional review boards: Australia: Human Research Ethics Committee (HREC 3) University of Sydney; Greece: Research Ethics Committee University of Crete; Poland: Komisja Bioetyczna przy Beskidziej Izble Lekarskiej – Bielsko Biala; Komisji Bioetycznej przy Śląskiej Izbie Lekarskiej; Silesian Medical Society (Śląska Izba Lekarska); Bioethics Committee at Lower Silesian Medical Association; Bioethics Committee at the Medical University of Biaystok; Portugal: North Health Regional Administration (ARS Norte); Matosinhos Local Health Unit (ULS Matosinhos); Guimarães Hospital; Center Health Regional Administration (ARS Centro); Regional Health Administration of Lisbon and Tagus Valley (ARS LVT); Spain: Comité de Ética de la Investigación (CEI) Islas Baleares; CEI Hospital Universitario de Gran Canaria; The Netherlands: Medisch Ethische Toetsingscommissie (METC) Assen exempted this study.

### Patient and public involvement

No patients were directly involved in the conceptualization and design of the study. A scientific advisory board has been set up to provide advice on the study protocol, the conduct of the study, data to be collected, statistical analysis and interpretation of the data. All members of the scientific advisory board are distinguished researchers and/or clinicians in the field of respiratory medicine and care for patients with COPD. For the contributing sites, the data collection raised awareness of the importance of a suboptimal PIF and/or inhalation technique errors in COPD patients and their medication adherence rates. Likewise, the participants could receive inhalation technique instructions during the visit. We plan on sharing our findings with clinicians, patients, and the public.

### Reporting summary

Further information on research design is available in the Nature Research Reporting Summary linked to this article.

## Results

### Study population

A total of 1434 participants with COPD from Australia, Greece, the Netherlands, Poland, Portugal and Spain were included in the study (Supplementary Table 5) and provided signed informed consent. Supplementary Fig. 1 shows a flow chart of the selection of the study population. Of these participants, 50.1% were female and the mean (SD) age was 69.2 (9.3) years. COPD severity was available for 801 participants and classified as GOLD stage I in 23.6%, II in 54.9%, III in 17.4% and IV in 4.1%. Participant characteristics are shown in Table 1.

**Table 1:** Overview of participant characteristics.

		PIF optimal (*n* = 987)	PIF suboptimal (*n* = 402)	Total (*n* = 1434)	*P* value
Female	*n* (%)	493 (49.9)	212 (52.7)	718 (50.1)	0.346
Age (years)	Mean (SD)	68.6 (9.2)	70.9 (9.3)	69.2 (9.3)	<0.001
GOLD stage	*n* (% non-missing)	551 (55.8)	209 (52.0)	801 (55.9)	0.959
	I, *n* (%)	131 (23.8)	49 (23.4)	189 (23.6)	
	II, *n* (%)	308 (55.9)	114 (54.5)	440 (54.9)	
	III, *n* (%)	91 (16.5)	38 (18.2)	139 (17.4)	
	IV, *n* (%)	21 (3.8)	8 (3.8)	33 (4.1)	
Years since COPD diagnosis	*n* (% non-missing)	974 (98.7)	398 (99.0)	1417 (98.8)	0.481
	Median (IQR)	8.0 (5.0;14.0)	7.0 (4.0;14.0)	8.0 (5.0;14.0)	
Body mass index (kg/m^2^)	*n* (% non-missing)	986 (99.9)	402 (100.0)	1433 (99.9)	0.016
	<18.5, *n* (%)	15 (1.5)	7 (1.7)	22 (1.5)	
	18.5–<25, *n* (%)	279 (28.3)	145 (36.1)	432 (30.1)	
	≥25–<30, *n* (%)	388 (39.4)	148 (36.8)	562 (39.2)	
	≥30–<40, *n* (%)	283 (28.7)	89 (22.1)	382 (26.7)	
	≥40, *n* (%)	21 (2.1)	13 (3.2)	35 (2.4)	
Smoking status	Current, *n* (%)	307 (31.1)	119 (29.6)	436 (30.4)	<0.001
	Former, *n* (%)	583 (59.1)	213 (53.0)	824 (57.5)	
	Never, *n* (%)	97 (9.8)	70 (17.4)	174 (12.1)	
Medication class in the primary inhaler	LABA, *n* (%)	84 (8.5)	25 (6.2)	112 (7.8)	<0.001
	LAMA, *n* (%)	265 (26.8)	112 (27.9)	385 (26.8)	
	LABA/LAMA, *n* (%)	270 (27.4)	71 (17.7)	357 (24.9)	
	LABA/LAMA/ICS, *n* (%)	31 (3.1)	26 (6.5)	63 (4.4)	
	ICS, *n* (%)	6 (0.6)	3 (0.7)	9 (0.6)	
	ICS/LABA, *n* (%)	331 (33.5)	163 (40.5)	506 (35.3)	
	Short-acting, *n* (%)	0 (0.0)	2 (0.5)	2 (0.1)	
Complete medication regimen	LAMA or LABA or ICS mono, *n* (%)	234 (23.7)	86 (21.4)	325 (22.7)	0.002
	LAMA + LABA, *n* (%)	264 (26.7)	78 (19.4)	359 (25.0)	
	ICS + (LAMA or LABA), *n* (%)	284 (28.8)	123 (30.6)	419 (29.2)	
	Triple therapy, *n* (%)	205 (20.8)	115 (28.6)	331 (23.1)	
Cardiovascular comorbidity	*n* (% non-missing)	982 (99.5)	399 (99.3)	1426 (99.4)	0.022
	*n* (%)	426 (43.4)	200 (50.1)	642 (45.0)	
Comorbid asthma	*n* (%)	165 (16.7)	71 (17.7)	246 (17.2)	0.671
Clinical COPD Questionnaire (CCQ)	Mean (SD)	1.7 (1.0)	1.9 (1.1)	1.7 (1.1)	<0.001
Exacerbations, moderate (*n*, %)	0, *n* (%)	785 (79.5)	299 (74.4)	1113 (77.6)	0.115
	1, *n* (%)	111 (11.2)	48 (11.9)	167 (11.6)	
	2, *n* (%)	41 (4.2)	29 (7.2)	72 (5.0)	
	3, *n* (%)	23 (2.3)	12 (3.0)	37 (2.6)	
	≥4, *n* (%)	27 (2.7)	14 (3.5)	45 (3.1)	
Exacerbations, severe (*n*, %)	0, *n* (%)	962 (97.5)	381 (94.8)	1386 (96.7)	0.047
	1, *n* (%)	20 (2.0)	16 (4.0)	38 (2.6)	
	2, *n* (%)	2 (0.2)	3 (0.7)	5 (0.3)	
	3, *n* (%)	2 (0.2)	0 (0.0)	2 (0.1)	
	≥4, *n* (%)	1 (0.1)	2 (0.5)	3 (0.2)	

**Fig. 2:**
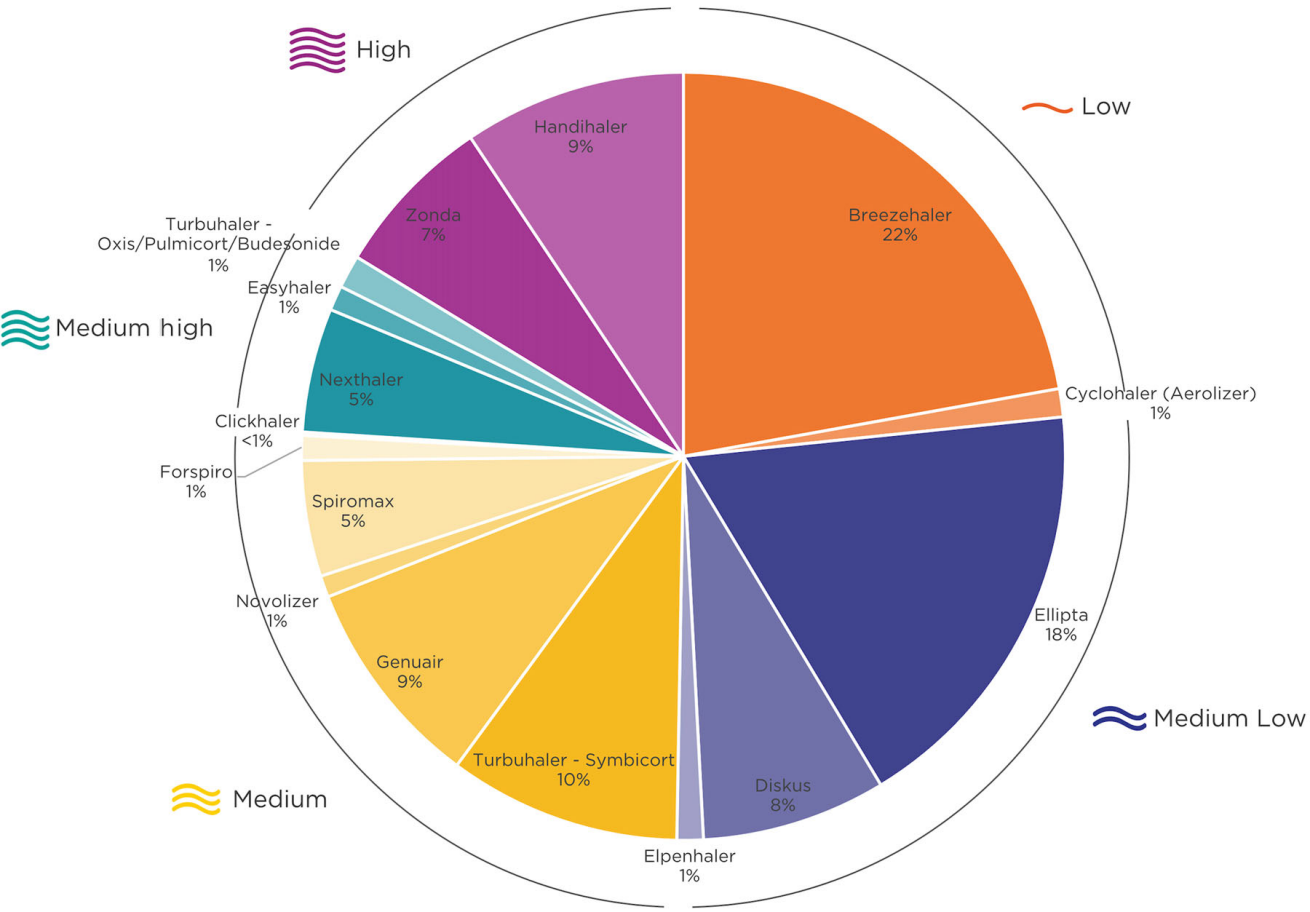
Distribution of the inhaler resistance clusters and inhaler type at the moment of study visit. Primary inhaler of each participant (*n* = 1434) is shown.

The primary inhaler used by the participants at the study visit are illustrated in Fig. 2. DPIs with a medium and medium-low internal resistance made up for over half (54%) of the DPIs used in the study. The majority of participants (66.2%) had received their last inhalation instruction more than a year prior to study entry, and 16% had never received an instruction on how to use their inhaler.

Mean (SD) CCQ-score and CAT-score were 1.7 (1.1) and 13.6 (7.8), respectively. In the previous 12 months, there were a total of 680 moderate and 77 severe exacerbations reported in 331 participants.

### Critical inhalation errors and adherence

**Fig. 3:**
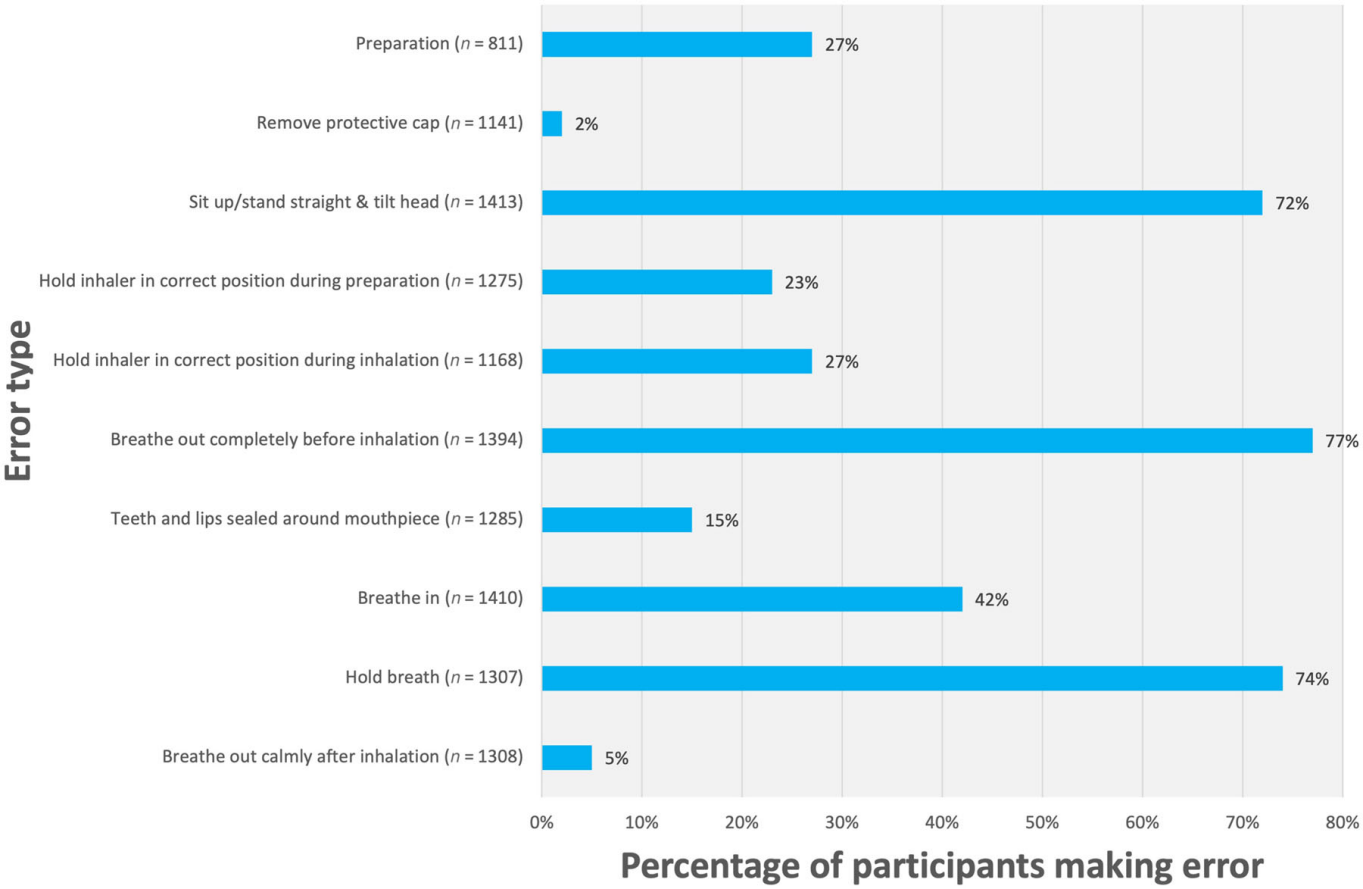
Frequency of observed inhalation technique errors. Inhalation technique was evaluated by grouping errors into distinct categories (*y-axis*), and the percentage of participants in this study making these errors are presented (*x-axis*).

The frequency of observed inhalation technique errors in this study is shown in Fig. 3.

Three inhalation technique errors were associated with CCQ and were thus identified as critical errors: (1) ‘teeth and lips sealed around mouthpiece’ (*p* = 0.093), (2) ‘breathe in’ (*p* = 0.001) and (3) ‘breathe out calmly after inhalation’ (*p* = 0.093). There was no significant relation between inhalation technique errors and exacerbations. Inhalation technique errors were common with a total of 4792 errors observed: 17.6% of these were a critical error (*n* = 846). Over half of the participants (51.7%) made one or more critical error.

Medication adherence was limited in this study population, as measured with modified TAI: 73.6% was classified as non-adherent. A total of 39.4% was classified as sporadic non-adherent, 25.5% as deliberate non-adherent and 53.7% as unconscious non-adherent.

### Associations of PIF, inhalation technique errors, adherence and health status

**Fig. 4:**
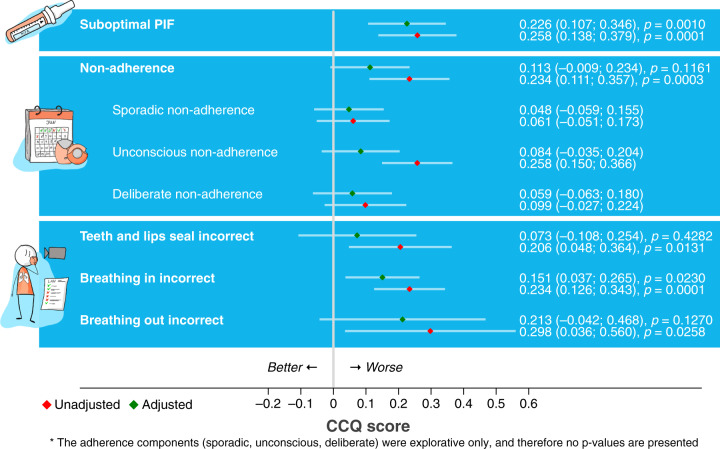
Factors associated with health status. Associations between PIF, adherence, critical inhalation errors and CCQ as a measure of health status with and without adjusting for confounders.

A suboptimal PIF (0.226 (95% CI 0.107–0.346, *p* = 0.001) and the critical error ‘breathe in’ (0.151 (95% CI 0.037–0.265, *p* = 0.023) were associated with worse CCQ-score after correcting for confounders. Adherence and other inhalation technique errors were not significantly associated with this measure of health status (Fig. 4).

### Associations of PIF, inhalation technique errors, adherence and exacerbations

**Fig. 5:**
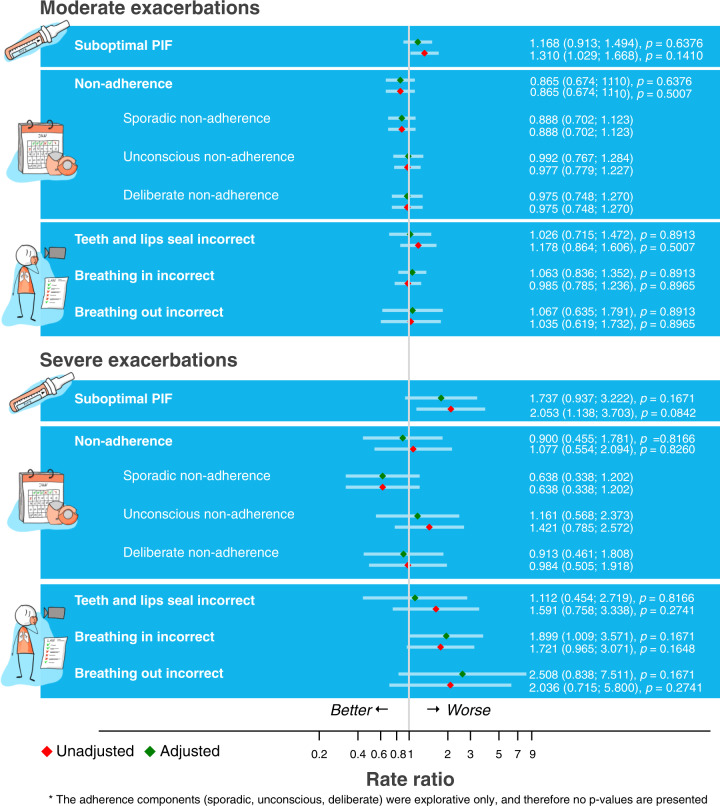
Factors associated with exacerbations. Associations between PIF, inhalation errors, adherence and moderate or severe exacerbations.

No significant association was found between moderate and severe exacerbations in the prior 12 months and suboptimal PIF after correcting for multiple testing (Fig. 5). Furthermore, adherence and inhalation errors were also not associated with the number of moderate or severe exacerbations (Fig. 5).

### Peak inspiratory flow (PIF) and inhaler resistance

The proportion of participants with a suboptimal PIF did not increase with the internal resistance of the inhaler (Supplementary Fig. 2). There was, however, a difference per inhaler resistance cluster and the three identified critical errors (Supplementary Fig. 3). The error ‘teeth and lips sealed around mouthpiece’ was most frequently made by participants with a medium-low resistance device. On more detailed inspection, it was found that this error was almost solely made by participants using the Ellipta device. It was observed that users of the Ellipta inhaler often opened the protective cap to the incorrect side, before putting the inhaler between the lips. When opening the protective cap to the incorrect side, the air vents of the Ellipta point downwards instead of the correct upwards position, which may lead to medication loss via these air vents. Therefore, this step was often scored as being incorrect for Ellipta inhaler users.

The error ‘breathe in’ was most frequently made by participants with a low resistance device. The error ‘breathe out calmly after inhalation’ was most frequently made by participants with a low resistance device.

**Fig. 6:**
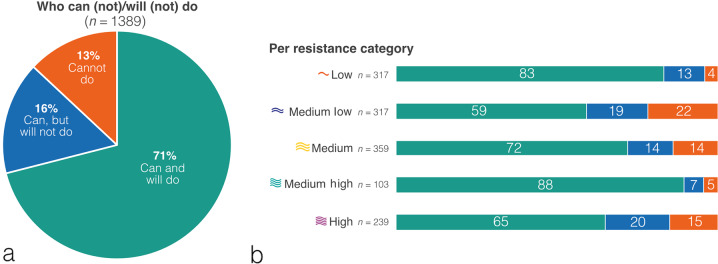
Overview of participants. Participants who can (not)/will (not) reach optimal PIF (**a**), and percentage of participants who can (not)/will(not) do per inhaler resistance category (**b**). Peak Inspiratory Flow categories based on In-Check DIAL G-16, by internal device resistance category.

Figure 6 shows which inhaler resistances were used by the three patient groups: ‘can do and will do’, ‘can do, but will not do’ and ‘cannot do’. This illustrates that for each inhaler resistance there is a proportion of users who cannot generate sufficient inspiratory flow for their device.

## Discussion

The PIFotal study demonstrated that a suboptimal PIF is associated with worse health status. Nearly a third of participants (29%) did not generate an optimal PIF for their own device during a typical inhalation manoeuvre, suggesting that PIF may be a critical inhaler factor not to be overlooked when selecting an inhaler device. Furthermore, the considerable proportion (16%) of participants in the ‘can, but will not do’ group, i.e. participants who were able to generate an optimal PIF but failed to do so, indicates that there is potential for interventions targeting PIF such as by improving inhalation technique. In addition, 13% of participants were unable to produce a sufficient PIF, indicating healthcare professionals should consider selecting different inhalers (with lower internal resistance or inhalers with zero internal resistance such as pMDIs or SMIs) in these cases. A more detailed breakdown of the alternative DPIs that are available for participants who are currently on a low resistance device is provided in Supplementary Table 6. A graphical overview of the findings in the PIFotal study can be found in Supplementary Fig. 4.

No significant relation was found between moderate and severe exacerbations and sPIF. However, participants with suboptimal PIF on average had a 73% higher severe exacerbation rate compared with participants with optimal PIF (*p* = 0.167). This finding is in line with the literature for patients discharged from hospital following a disease exacerbation and indicates the need to assess whether a patient’s PIF is satisfactory at every given opportunity, to prevent a sustained worsening of the patient’s health status.

The proportion of participants with a suboptimal PIF does not increase with increasing resistance of the inhaler.

This might appear to be counter intuitive. Our video analysis showed that a proportion of participants inhaled too forcefully especially with low resistance inhalers, which can potentially lead to an unwanted higher drug deposition in the oropharynx and potentially greater oral bioavailability affecting the therapeutic index^30^. Another explanation could be that participants feel more resistance when using the high resistance devices and therefore put more effort in their inhalation to overcome the resistance. They feel they need to “work” to inhale their medication and consequently do so.

Adherence to inhaled medication has been shown to be an important factor in disease outcomes^31^, and in our study, this indeed appeared to be related to CCQ scores and exacerbations. After correcting the associations for potential confounders, these associations did not continue to be significant. An explanation could be that we assessed adherence cross-sectionally and related this to disease outcomes. The nature of this study does not allow to describe causality. Another explanation could be that the assessment of adherence using the TAI is not specific enough, as currently electronic monitoring is considered the gold standard. However, one could discuss that the TAI has been validated and has been proven related to outcomes. Nevertheless, these studies did not correct for the breadth of variables we have been able to do in PIFotal. Therefore, the relations between (different types) of adherence need to be studied further. Response bias may also have played a role as those who were completely non-adherent despite a poor disease status are expected to show lower response rates. This study highlights that there is a complex relation between adherence and outcomes, and future adherence studies should measure potential confounders carefully to be able to disentangle the specific contribution of adherence to outcomes.

This study assessed inhaler technique errors in over 1400 participants with COPD using video recording and independent scoring. We assessed all inhaler errors and linked them to outcomes. Previous studies in COPD used predefined critical errors based on literature or expert opinion^16^. The errors reported in the large COPD study by Molimard et al. showed some overlapping errors to be related to outcomes, but there were also differences^16^. Our study did not highlight the importance of correct exhalation before inhalation, as was the case in Molimard’s study. If a patient does not exhale fully, forceful inhalation might not be possible due to lack of the inhaled volume^30^. In our study, this theoretical error was not related to outcomes. This warrants further discussion on the matter of which errors are actual critical errors. Further, future research needs to consider the role of patient perceptions and habits in using their device, on their actual technique, i.e. whether they feel, from experience that they need to use a deep and forceful breath or not when using their inhaler. It should not be underestimated that there is in fact a different sensation that participants experience in using devices of different internal resistance.

The findings in PIFotal complement previous findings on inhalation technique^32^, particularly from the CRITIKAL asthma study, emphasizing the importance of targeting inhaler training to reduce key critical errors^22^. This seems confirmed by a very recent publication, demonstrating that the incorporation of PIF-guided inhalation therapy into COPD treatment plan could reduce the risk of severe acute exacerbation^33^.

The strengths of PIFotal include the real-world design carried out in multiple countries and including a large group of participants with COPD.

We cannot exclude residual confounding, although our analyses were adjusted for all selected potential confounders based on literature and clinical expertise. One of the anticipated biases was the bias by indication for ICS-containing medication for those participants who experience exacerbations. We cannot exclude indication bias to have played a role either. Those with worse disease status may have received more ICS-containing regimes leading to device selection. However, sensitivity analyses showed that the association between a suboptimal PIF and CCQ was most accentuated and statistically significant in participants receiving triple therapy through an Ellipta inhaler (0.705 (0.175–1.235, *p* = 0.0092) (Supplementary Fig. 5), which supports our findings, although effect modification by full medication regimen for suboptimal PIFR on CCQ was not significant (*p* = 0.4186) (Supplementary Table 7).

Significant associations were found between suboptimal PIF and health status. It is, however, worth emphasizing that because of the current research design, the relations should be interpreted with caution, as they may not have a causal character.

Another limitation is that all measurements were performed in different countries. Although the multinational approach is very much a strength of PIFotal, it seems possible that inhalation instructions differ between countries. Although all investigators confirmed that the instruction for a maximal PIF was ‘for participants to do the very best they can’, and all investigators confirmed that this message had come across to all study participants, it is possible that small semantic differences in the instructions have occurred.

One could challenge our statistical method of multiple imputation to handle missing data. Fortunately, there was only missing data on PIF in 3.1% of the cases, implying that the risks accompanying multiple imputation were small.

Although it is not a limitation, it is worth emphasizing that this study was performed during the COVID-19 pandemic. This may have influenced some of the outcomes, for instance, the rate of exacerbations may not be entirely representative for a regular non-pandemic year^34^, and may also present a bias in terms of participants willing to partake in research at this time.

In this study, suboptimal PIF was significantly associated with poorer health status. It highlights the importance of selecting the optimal device for the patient, and PIF being a key parameter in this device selection. There was a trend but no significant association of sPIF and exacerbations. PIF is an important factor not to be overlooked when prescribing a DPI to COPD patients. The proportion of participants with a suboptimal PIF (29%) stresses the importance of taking PIF into account when selecting an DPI inhaler device. This is especially the case in the 13% of participants who cannot generate a sufficient inspiratory flow. As suboptimal PIF is shown to be associated with poorer health status, it is deemed valuable to assess whether PIF is sufficient at every given opportunity, as by doing so, healthcare professionals may be able to prevent sustained worsening of patients’ health status. Further research may elucidate which interventions targeting PIF are of use in selecting inhalation medication devices for COPD patients.

## Supplementary information

Supplementary material

REPORTING SUMMARY

## Data Availability

The data that support the findings of this study are available on request from the corresponding author J.K.

## Code availability

The custom code that is deemed central to the findings and conclusion in this manuscript are available on request from the corresponding author J.K.
