# Supplementary material for: Factors associated with health status and exacerbations in COPD maintenance therapy with dry powder inhalers
Source: NPJ Prim Care Respir Med. 2022 May 26;32:18. doi: 10.1038/s41533-022-00282-y (PMC9135702; doi:10.1038/s41533-022-00282-y)
Supplement: Supplementary file 1 — Supplementary material [file 41533_2022_282_MOESM1_ESM.pdf]

Supplementary information

Supplementary Table 1. Overview of DPIs included in the study, the assessment priority for this study and which PIF is required and optimal for effective inhalation.

Inhaler type	Assessment priority	Minimal PIF required (L/min)	Optimal PIF (L/min)
Ellipta®	1	30	60 [1,2]
Turbuhaler®	2	30	60 [1,2,3]
Breezhaler®	3	50	50 [1,2]
Zonda®	4	20	39 [4]
Genuair®	5	40	45 [1,2]
Novolizer®	6	35	50 [1,2]
Spiromax®	7	40	40 [1,2]
Diskus®	8	30	60 [1,2,3,5]
Handihaler®	9	20	30 [1,2]
Nexthaler®	10	35	35 [1,2]
Cyclohaler®	11	40	65 [1]
Easyhaler®	12	30	30 [1,2]
Forspiro®	13	30	60 [1,2]
Elpenhaler®	14	30	60 [6]
Clickhaler®	15	15	15 [7]

[1] Ghosh, S., Pleasants, R. A., Ohar, J. A., Donohue, J. F. & Drummond, M. B. Prevalence and factors associated with suboptimal peak inspiratory flow rates in COPD. Int. J. COPD 14, 585–595 (2019).
[2] van der Palen, J. Peak inspiratory flow through Diskus and Turbuhaler, measured by means of a peak inspiratory flow meter (In-Check DIAL ®). Respir. Med. (2003) doi:10.1053/rmed.2003.1289.
[3] Clark, A. R., Weers, J. G. & Dhand, R. The Confusing World of Dry Powder Inhalers: It Is All About Inspiratory Pressures, Not Inspiratory Flow Rates. J. Aerosol Med. Pulm. Drug Deliv. 33, 1–11 (2020).
[4] Correspondence with manufacturer TEVA, Nov 18 (2020).
[5] Virchow, J. C., Weuthen, T., Harmer, Q. J. & Jones, S. Identifying the features of an easy-to-use and intuitive dry powder inhaler for asthma and chronic obstructive pulmonary disease therapy: Results from a 28-day device handling study, and an airflow resistance study. Expert Opin. Drug Deliv. (2014) doi:10.1517/17425247.2014.949236.
[6] Correspondence with Paul Hagedoorn, Head of Inhalation Research Laboratory, RUG.
[7] Newhouse MT, Nantel NP, Chambers CB, Pratt B, Parry-Billings M. Clickhaler (a novel dry powder inhaler) provides similar bronchodilation to pressurized metered-dose inhaler, even at low flow rates. Chest. 115(4), 952–6 (1999).

Supplementary Table 2. Overview of inhaler step categories, adjusted from LAN. Netherlands Lung Alliance (www.inhalatorgebruik.nl)

	Category of inhalation steps
-	Emptying the mouth before starting the inhalation
-	Removing the protective cap
-	Preparation of the device
-	Holding inhaler in the correct position during preparation
-	Sitting up/standing straight & tilting head slightly backwards
-	Breathe out completely to empty lungs before inhalation (not into device)
-	Holding inhaler in the correct position during the inhalation
-	Sealing teeth and lips around the mouthpiece (and holding on to air vents correctly without obstructing air vents)
-	Inhaling either strong and deep, or calm and deep
-	Holding breath (at least 6 seconds or for as long as comfortable)
-	Breathe out calmly after the inhalation

Supplementary Table 3. Overview of confounder candidates for the different associations

All models:

- Country of residence
- Age
- Sex
- Body Mass Index
- Smoking status
- Educational level
- Medication class
- SARS-COV-2 history
- Lung comorbidity
- Cardiovascular comorbidity
- Depression
- Anxiety
- Diabetes mellitus
- Medication regimen

Specific for predictor *Suboptimal PIF*:

- Non-adherence
- All inhalation errors (except error related to PIF: ’Breathing in incorrect’)

Specific for predictor *Non-adherence*:

- All inhalation errors (except the critical errors that are included in the definition)

Specific for *Sporadic, Deliberate and Unconscious non-adherence*:

- Suboptimal PIF
- All inhalation errors

Supplementary Table 4. Overview of confounders included in the models

Outcome	Predictor	Identified confounders
CCQ	Suboptimal PIF	Medication regimen; Country of residence;
	Non-adherence	Anxiety; Country of residence; Depression; Error: Holding breath; Lung comorbidity; Device resistance;
	Sporadic non-adherence	Medication regimen; Cardiovascular comorbidity; Country of residence; Depression; Diabetes; Sex; Medication class; PIF insufficiency; Smoking status;
	Unconscious non-adherence	Medication regimen; Anxiety; Country of residence; Depression; Diabetes; Error: Preparation; Error: Breathing out after inhalation; Error: Teeth and lips sealed; Error: Holding breath; Sex; Lung comorbidity; Medication class; PIF insufficiency;
	Deliberate non-adherence	Medication regimen; Anxiety; BMI; Cardiovascular comorbidity; Depression; Diabetes; Error: Holding breath; Sex; Medication class; PIF insufficiency; Device resistance; Smoking status;
	Critical errors	Anxiety; Country of residence; Diabetes; Error: Preparation; Error: Breathing out after inhalation; Error: Breathing in; Sex; Lung comorbidity; Medication class; Device resistance; Smoking status;
Moderate exacerbations	Suboptimal PIF	Medication regimen; Country of residence;
	Non-adherence	-
	Sporadic non-adherence	-
	Unconscious non-adherence	Error: Teeth and lips sealed; Device resistance;
	Deliberate non-adherence	-
	Critical errors	Error: Preparation; Error: Breathing out after inhalation; Error: Breathing in; Device resistance;
	Suboptimal PIF	Error: Holding breath; Lung comorbidity; Device resistance;

Severe exacerbations	Non-adherence	Anxiety; Error: Preparation;
	Sporadic non-adherence	-
	Unconscious non-adherence	Anxiety; Country of residence; Error: Preparation; Error: Breathing out after inhalation; Error: Remove protective cap; Error: Teeth and lips sealed; Lung comorbidity; Medication class; PIF insufficiency;
	Deliberate non-adherence	Anxiety; PIF insufficiency; Device resistance;
	Critical errors	Medication regimen; Country of residence; Error: Preparation; Error: Breathing out after inhalation; Error: Breathing in; Lung comorbidity; Device resistance;

Supplementary Figure 1. Flowchart of study population selection.

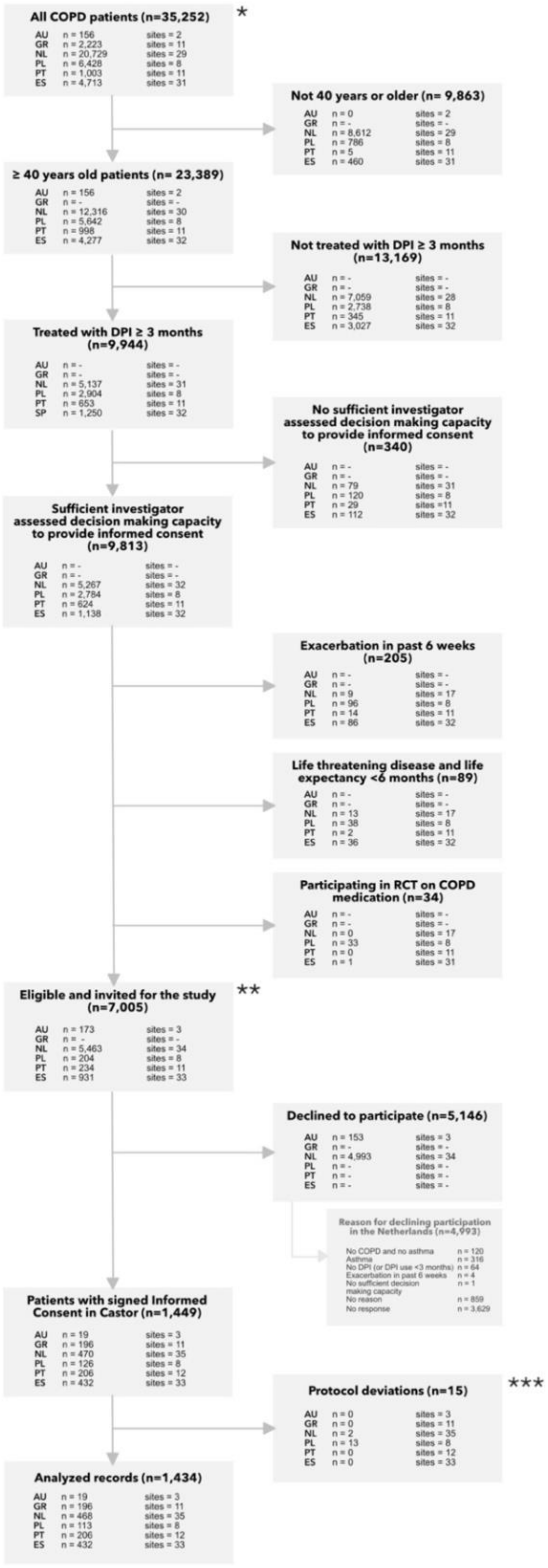

** The data provided by the community pharmacies from Spain are estimations. The data from Greece are also estimations and were calculated before the COVID-19 pandemic. Three sites in Portugal reported relatively low numbers of COPD patients as the sites are specialized care units.*
*** Not all eligible patients in Spain, Portugal and Poland were invited to participate due to time constraints.*
**** These records were archived because the measurements deviated from the study protocol.*

Supplementary Figure 2. Typical PIF and inhaler resistance clusters.

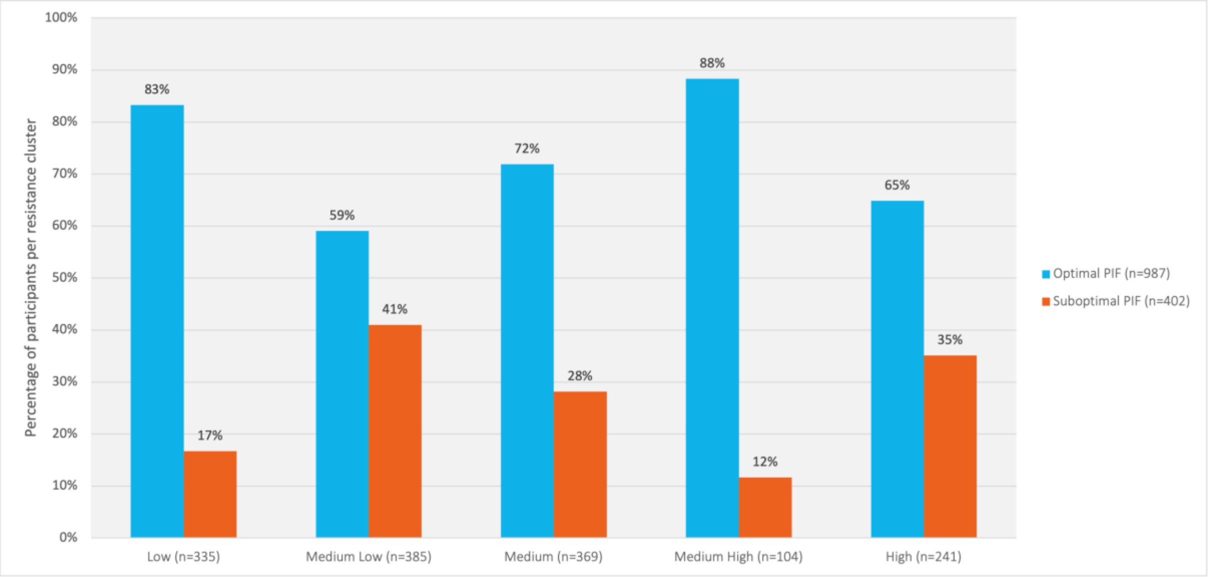

Supplementary Figure 3. Inhalation technique errors by inhaler resistance clusters.

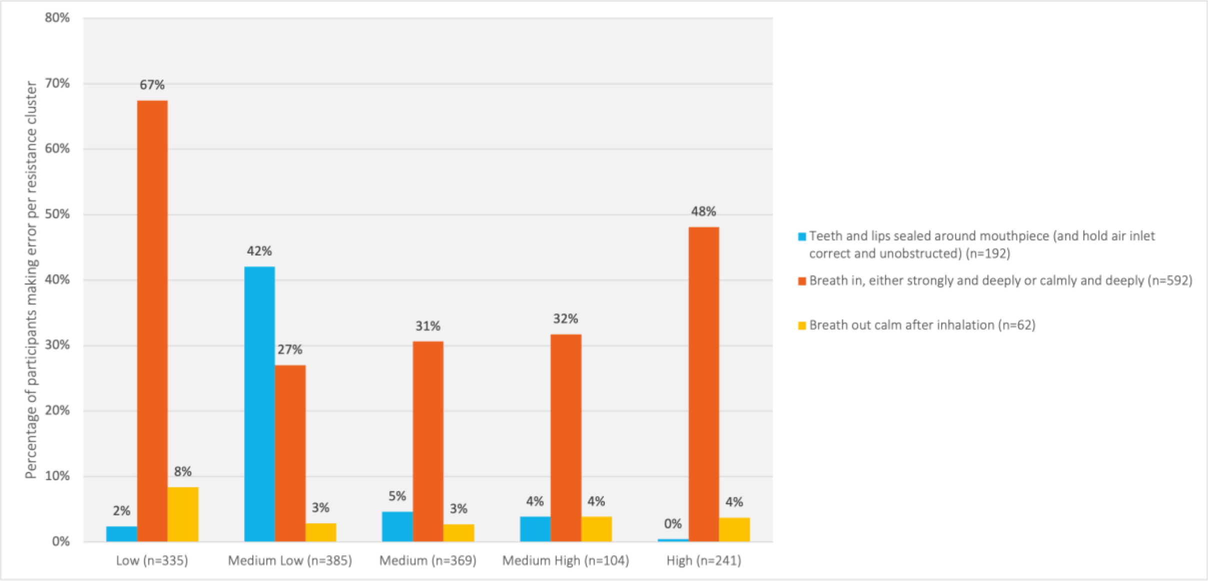

Supplementary Figure 4. Graphical overview of the PIFotal study findings.

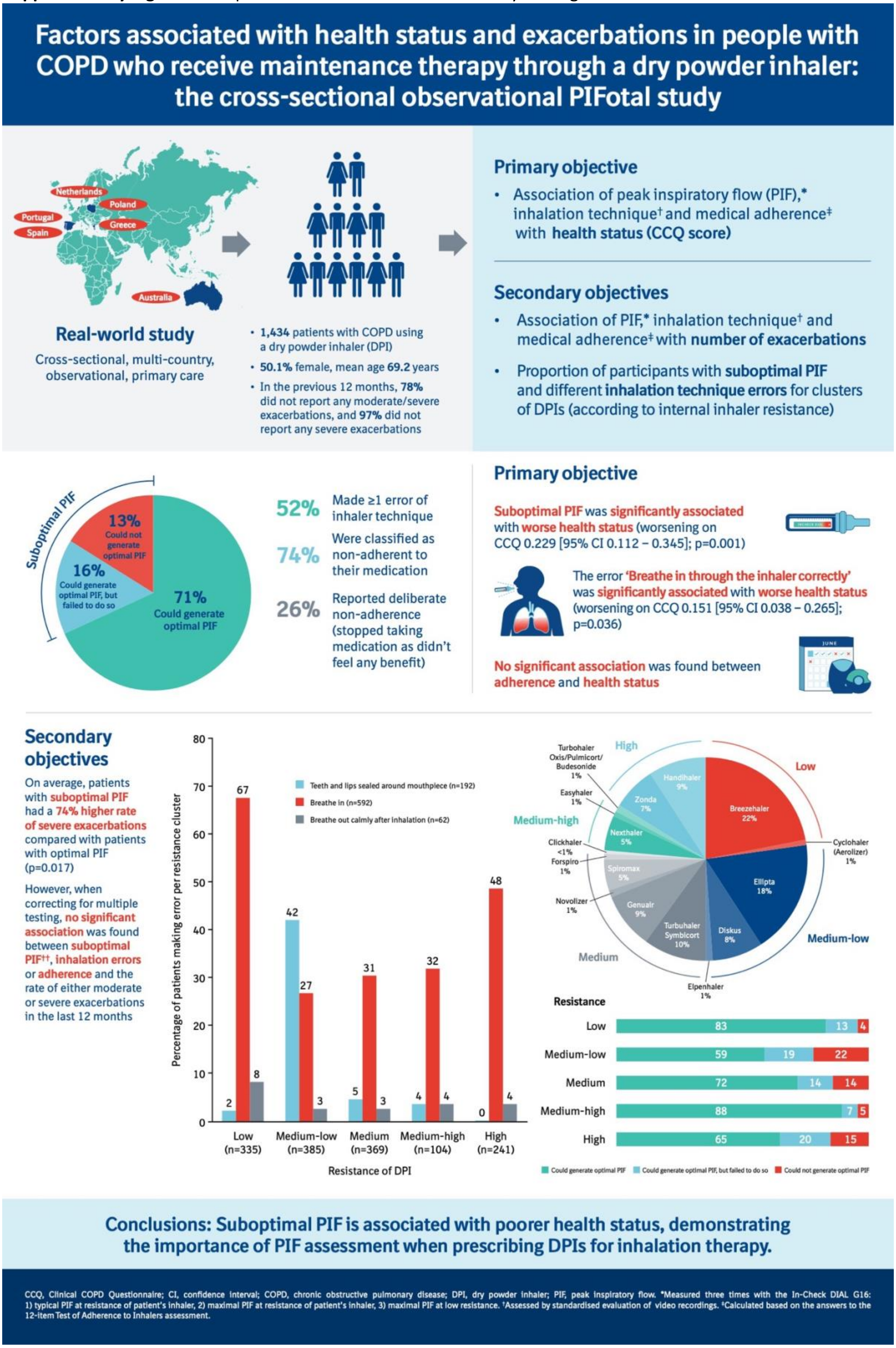

Supplementary Table 5. Overview of included participants per country. There were 1,434 participants in the study from 6 countries, 102 study sites and 621 different GP practices

Country	N (%) participants
Australia	19 (1.3)
Greece	196 (13.7)
Netherlands	468 (32.6)
Poland	113 (7.9)
Portugal	206 (14.4)
Spain	432 (30.1)
Total	1,434

Supplementary Table 6. Participants for whom an alternative DPI with lower resistance is available.

Medication class	Number of participants who 'cannot do'	N (%) for whom there is an alternative DPI with which they could do	
		Exists and is available	With same substance(s)
LABA	10	10 (100)	7 (70)
LAMA	49	48 (98)	44 (90)
LABA/LAMA	26	22 (85)	16 (62)
LABA/LAMA/ICS	6	3 (50)	3 (50)
ICS	3	3 (100)	1 (33)
ICS/LABA	87	87 (100)	80 (92)
Short-acting	2	2 (100)	2 (100)
Total	183	175 (96)	153 (84)

This table shows that for 96% of the participants there exists an alternative DPI for their medication class with a resistance below the patient's maximal PIF. There were fewest alternatives for participants using a LABA/LAMA combination inhaler or a triple therapy inhaler. All of the identified alternative DPIs were also available in the participants' countries of residence. Of the 175 alternative devices, 153 (84%) also matched the inhalation substance(s), so that a switch to a lower resistance device without switching medication is possible.

Supplementary Figure 5. Medication class specific effects through effect modification on PIF sufficiency on CCQ

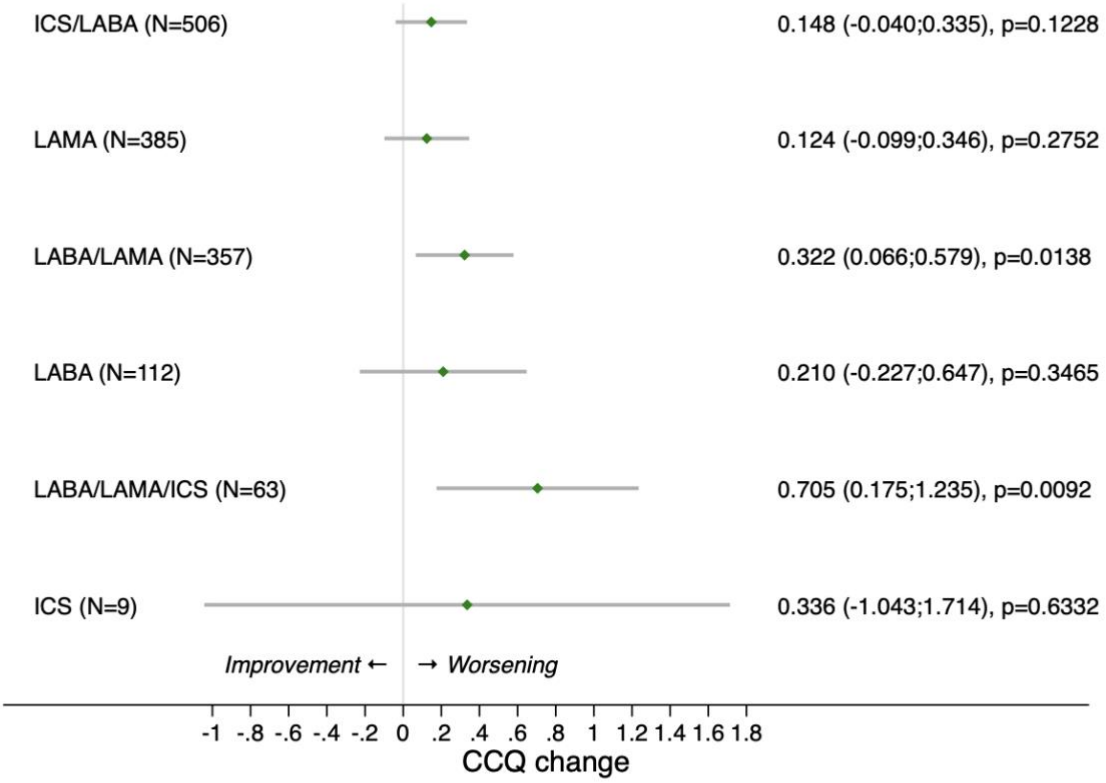

Supplementary Table 7. Effect modification by full medication regimen for suboptimal PIFR on CCQ – model results

	Unadjusted		Adjusted		
Predictor	Effect size (95% CI)	P-value	Effect size (95% CI)	P-value	Included confounders
a Suboptimal PIFR	0.228 (-0.003;0.459)	0.0529	0.194 (-0.030;0.419)	0.0902	Medication class; Depression; Country of residence; BMI
b Medication regimen					
Triple therapy	Reference		Reference		
ICS + (LAMA or LABA)	-0.338 (-0.523;-0.152)	0.0004	-0.137 (-0.349;0.075)	0.2056	
LAMA + LABA	-0.167 (-0.353;0.019)	0.0776	-0.103 (-0.369;0.164)	0.4499	
LAMA or LABA or ICS	-0.364 (-0.556;-0.172)	0.0002	-0.518 (-0.735;-0.302)	<0.0001	
Interaction a x b		0.6178		0.4186	
Triple therapy	Reference		Reference		
ICS + (LAMA or LABA)	-0.083 (-0.397;0.232)	0.6056	-0.104 (-0.408;0.199)	0.5008	
LAMA + LABA	0.110 (-0.240;0.460)	0.5372	0.149 (-0.187;0.486)	0.3839	
LAMA or LABA or ICS	0.103 (-0.236;0.443)	0.5511	0.097 (-0.231;0.424)	0.5622	

Unit is average difference in CCQ score
