# Supplementary material for: Factors associated with health status and exacerbations in COPD maintenance therapy with dry powder inhalers
Source: NPJ Prim Care Respir Med. 2022 May 26;32:18. doi: 10.1038/s41533-022-00282-y (PMC9135702; doi:10.1038/s41533-022-00282-y)
Supplement: Supplementary file 2 — REPORTING SUMMARY [file 41533_2022_282_MOESM2_ESM.pdf]

nature portfolio

Corresponding author(s): Professor Janwillem Kocks

Last updated by author(s): Mar 11, 2022

Reporting Summary

Nature Portfolio wishes to improve the reproducibility of the work that we publish. This form provides structure for consistency and transparency in reporting. For further information on Nature Portfolio policies, see our Editorial Policies and the Editorial Policy Checklist.

Statistics

For all statistical analyses, confirm that the following items are present in the figure legend, table legend, main text, or Methods section.

n/a	Confirmed	
☐	☒	The exact sample size (n) for each experimental group/condition, given as a discrete number and unit of measurement
☐	☒	A statement on whether measurements were taken from distinct samples or whether the same sample was measured repeatedly
☐	☒	The statistical test(s) used AND whether they are one- or two-sided<br>*Only common tests should be described solely by name; describe more complex techniques in the Methods section.*
☐	☒	A description of all covariates tested
☐	☒	A description of any assumptions or corrections, such as tests of normality and adjustment for multiple comparisons
☐	☒	A full description of the statistical parameters including central tendency (e.g. means) or other basic estimates (e.g. regression coefficient) AND variation (e.g. standard deviation) or associated estimates of uncertainty (e.g. confidence intervals)
☐	☒	For null hypothesis testing, the test statistic (e.g. F, t, r) with confidence intervals, effect sizes, degrees of freedom and P value noted<br>*Give P values as exact values whenever suitable.*
☐	☒	For Bayesian analysis, information on the choice of priors and Markov chain Monte Carlo settings
☐	☒	For hierarchical and complex designs, identification of the appropriate level for tests and full reporting of outcomes
☐	☒	Estimates of effect sizes (e.g. Cohen's d, Pearson's r), indicating how they were calculated

Our web collection on statistics for biologists contains articles on many of the points above.

Software and code

Policy information about availability of computer code

Data collection	No software was used for data collection.
Data analysis	N/A

For manuscripts utilizing custom algorithms or software that are central to the research but not yet described in published literature, software must be made available to editors and reviewers. We strongly encourage code deposition in a community repository (e.g. GitHub). See the Nature Portfolio guidelines for submitting code & software for further information.

Data

Policy information about availability of data

All manuscripts must include a data availability statement. This statement should provide the following information, where applicable:

- Accession codes, unique identifiers, or web links for publicly available datasets
- A description of any restrictions on data availability
- For clinical datasets or third party data, please ensure that the statement adheres to our policy

The data that support the findings of this stuady are available on request from the corresponding author JK.

Field-specific reporting

Please select the one below that is the best fit for your research. If you are not sure, read the appropriate sections before making your selection.

☒ Life sciences ☐ Behavioural & social sciences ☐ Ecological, evolutionary & environmental sciences

For a reference copy of the document with all sections, see nature.com/documents/nr-reporting-summary-flat.pdf

Life sciences study design

All studies must disclose on these points even when the disclosure is negative.

Sample size	A sample size calculation was performed prior to study execution. A sample size of 1,200 participants was estimated to be sufficient to achieve sufficient (≥80%) statistical power. It was assumed that the difference between optimal and suboptimal PIF would lead to a Clinical COPD Questionnaire (CCQ) score difference of 0.2 points.This minimal detectable difference yielded a sample size of 1,176 participants, with an alpha of 5% and a power of 80%.
Data exclusions	N/A
Replication	Reproducibility of study findings were tested by several senstivity analyses. All attempts at replication were succesful.
Randomization	Due to the cross-sectional observational design, randomization was not applicable.
Blinding	Blinding was not applicable due to the study design.

Reporting for specific materials, systems and methods

We require information from authors about some types of materials, experimental systems and methods used in many studies. Here, indicate whether each material, system or method listed is relevant to your study. If you are not sure if a list item applies to your research, read the appropriate section before selecting a response.

Materials & experimental systems

n/a	Involved in the study	
☒	☐	Antibodies
☒	☐	Eukaryotic cell lines
☒	☐	Palaeontology and archaeology
☒	☐	Animals and other organisms
☐	☒	Human research participants
☒	☐	Clinical data
☒	☐	Dual use research of concern

Methods

n/a	Involved in the study	
☒	☐	ChIP-seq
☒	☐	Flow cytometry
☒	☐	MRI-based neuroimaging

Human research participants

Policy information about studies involving human research participants

Population characteristics	Please see Table 1 of the manuscript for an overview of the population characteristics.
Recruitment	Patients were recruited via GPs and pharmacies in 6 countries (Australia, Poland, Portugal, Spain, Greece & The Netherlans)
Ethics oversight	The PIFotal COPD study protocol has received approvals from the following institutional ethics committees/institutional review boards: Australia: Human Research Ethics Committee (HREC 3) University of Sydney; Greece: Research Ethics Committee University of Crete; Poland: Komisja Bioetyczna przy Beskidziej Izble Lekarskiej – Bielsko Biala; Komisji Bioetycznej przy Śląskiej Izbie Lekarskiej; Silesian Medical Society (Śląska Izba Lekarska); Bioethics Committee at Lower Silesian Medical Association; Bioethics Committee at the Medical University of Biaystok; Portugal: North Health Regional Administration (ARS Norte); Matosinhos Local Health Unit (ULS Matosinhos); Guimarães Hospital; Center Health Regional Administration (ARS Centro); Regional Health Administration of Lisbon and Tagus Valley (ARS LVT); Spain: Comité de Ética de la Investigación (CEI) Islas Baleares; CEI Hospital Universitario de Gran Canaria; The Netherlands: Medisch Ethische Toetsingscommissie (METC) Assen exempted this study.

Note that full information on the approval of the study protocol must also be provided in the manuscript.
